# Impact of PPAR-Alpha Polymorphisms—The Case of Metabolic Disorders and Atherosclerosis

**DOI:** 10.3390/ijms20184378

**Published:** 2019-09-06

**Authors:** Massimiliano Ruscica, Marco Busnelli, Enrico Runfola, Alberto Corsini, Cesare R. Sirtori

**Affiliations:** 1 Department of Pharmacological and Biomolecular Sciences, Università degli Studi di Milano, 20122 Milan, Italy (M.B.) (A.C.); 2 Centro Dislipidemie, A.S.S.T. Grande Ospedale Metropolitano Niguarda, 20122 Milan, Italy (E.R.) (C.R.S.); 3 Multimedica, IRCCS, 20122 Milan, Italy

**Keywords:** PPARα, polymorphism L162V, polymorphism Val227Ala, rs4253778 G > C, rs4253776 A > G

## Abstract

Peroxisome proliferator activated receptor α (PPARα) has the most relevant biological functions among PPARs. Activation by drugs and dietary components lead to major metabolic changes, from reduced triglyceridemia to improvement in the metabolic syndrome. Polymorphisms of PPARα are of interest in order to improve our understanding of metabolic disorders associated with a raised or reduced risk of diseases. PPARα polymorphisms are mainly characterized by two sequence changes, L162V and V227A, with the latter occurring only in Eastern nations, and by numerous SNPs (Single nucleotide polymorphisms) with a less clear biological role. The minor allele of L162V associates with raised total cholesterol, LDL-C (low-density lipoprotein cholesterol), and triglycerides, reduced HDL-C (high-density lipoprotein metabolism), and elevated lipoprotein (a). An increased cardiovascular risk is not clear, whereas a raised risk of diabetes or of liver steatosis are not well supported. The minor allele of the V227A polymorphism is instead linked to a reduction of steatosis and raised γ-glutamyltranspeptidase levels in non-drinking Orientals, the latter being reduced in drinkers. Lastly, the minor allele of rs4353747 is associated with a raised high-altitude appetite loss. These and other associations indicate the predictive potential of PPARα polymorphisms for an improved understanding of human disease, which also explain variability in the clinical response to specific drug treatments or dietary approaches.

## 1. Introduction

Metabolic disorders underlying an enhanced risk of cardiovascular disease (CVD), i.e., diabetes and dyslipidemia, may be also associated with excess body weight, which frequently leads to the metabolic syndrome (MetS) [1]. While reduction in low density lipoprotein cholesterol (LDL-C) still remains a foremost target in cardiovascular (CV) prevention, low levels of high density lipoprotein cholesterol (HDL-C) and elevated triglycerides (TG) play an important and independent role and have become a major target of treatment [2]. Thus, the association between elevated small LDL, low HDL-C, and high TGs bring about “atherogenic dyslipidemia,” which markedly raises CV risk and for which different therapeutic strategies have been developed [3]. While treatment of hypercholesterolemia can be successfully handled with drugs targeting cholesterol biosynthesis [4], MetS is characterized by a number of diverse metabolic abnormalities that are more difficult to pursue. Control of the metabolic syndrome and atherogenic dyslipidemia may be achieved by the agonists of the family of peroxisome proliferator-activator receptors (PPARs), i.e., nuclear-hormone receptors and transcription factor activating ligands, discovered in the early 1990s [5], and potentially involved in the development of CVD and metabolic disorders such as diabetes and inflammatory diseases [6].

The activation of the PPARs may lead to metabolic responses potentially altering homeostasis [7]. The family of PPARs is comprehensive of PPARα, PPAR β/δ, and PPARγ [8]. Each of these is codified by different genes and shows overlapping interspecies sequences, particularly in the DNA binding domain (DBD) and ligand binding domain (LBD). The structure of PPARs is quite similar for all three members, and characterized by: (1) an N-terminal region, responsible for exerting activity and achieving phosphorylation, (2) a DBD consisting of two Zinc-fingers, one on PPAR, and one on (retinoid X receptor) RXR, with a linker region, (3) a flexible hinge region, (4) an LBD, and (5) the C-terminal region [9].

Different PPARs are encoded by different chromosomes. PPARα is coded by chromosome 22q12-13.1, PPAR β/δ by chromosome 6p21.2-21.1, and PPARγ by chromosome 3p25.2 [10]. PPARs are expressed in different tissues, α in liver, kidney, heart, muscle, and adipose tissue [11], β/δ in muscle, brain adipose tissue and skin, γ is expressed in three forms: γ1-virtually in all tissues including heart, muscle, colon, kidney, pancreas, and spleen, γ2 (30 amino acid longer than γ1) mainly in adipose tissue, and γ3 in macrophages, large intestine, and white adipose tissue [12]. While the two latter PPARs were identified at a later time, presenting close similarities and a limited scope of metabolic control and potential treatment approaches, PPARα has been more extensively investigated and acts on a wider range of metabolic steps, which are activated by a large number of agonists. For this purpose, by using Pubmed.gov, we revised available English-language studies published from January 1990 to August 2019 and relevant to the key clinical questions discussed in this review. Search terms included PPARα polymorphisms, metabolic syndrome, diabetes, non-alcoholic fatty liver disease (NAFLD), and atherosclerosis.

## 2. PPARα Expression and Function

PPARα expression is high in hepatocytes, enterocytes, and in vascular and immune cell types, such as monocytes/macrophages, smooth muscle cells, endothelial cells, lymphocytes, and non-neuronal CNS cells [13], i.e., microglia and astroglia [14]. All these cell types play a crucial role, particularly in the case of the liver, in fatty acid oxidation, which provides energy to peripheral tissues [15]. The lipid peroxisomal β-oxidation further occurs in brown adipose tissue and plays a potential role in oxidant/antioxidant pathways [16].

The activation of PPARα, particularly by drugs or nutraceuticals, exerts a central role in lipid and lipoprotein metabolism and may reduce the dyslipidemia associated with the MetS [17]. During the fasting state, PPARα is activated by adipose tissue-derived fatty acids, which enhance the production of ketone bodies through lipid oxidation in the liver and peripheral mononuclear cells [18]. The mechanism of metabolic activation for PPARα is by way of a complex pathway, through the formation of a heterodimer with the RXR. This determines a conformational change, which reflects gene transcription or transactivation [19]. By this mechanism, the inhibited expression of nuclear factor KB (NF-kB) or activator protein 1 responsible for inflammation [20] may take place. Interaction of activated PPARα with response elements occurs by binding to a specific DNA sequence called PPAR Response Element (PPRE), located upstream of the target gene, and by binding to another cofactor, which leads to an active transcriptional complex followed by gene transcription [21].

The complexity of this translational mechanism involving phosphorylation, acetylation, and ubiquitination, through the action of the c-Jun terminal kinase and (5’ AMP-activated protein kinase) AMPK protein kinase 1 and 2, protein kinase A, and synthase kinase [22], makes the process very vulnerable to any abnormality in DNA sequences, which makes evaluation of eventual SNPs very significant.

## 3. PPARα-Metabolic Effects and Identified Agonists

Activity of PPARα is mainly of a catabolic type, particularly activating oxidative mechanisms leading to enhanced energy provision. Agonists of PPARα can be endogenous and synthetic. Among endogenous activators are unsaturated fatty acids, eicosanoids [23], epoxyeicosatrienoic acids, leukotriene B4, prostaglandins, and oleoylethanolamide (OEA) [24].

PPARα activators appear to belong to a general class of “fraudulent fatty acids,” i.e., agents that do not undergo full mitochondrial catabolism. Instead, they need exposure to peroxisomes for their complete catabolism [25]. These dietary and synthetic compounds frequently determine peroxisomal proliferation, particularly in rodents, but not in higher species [26].

Synthetic drugs that can bind mainly to PPARα (in some cases, to multiple isoforms) belong again to the fraudulent fatty acid series, particularly fibrates (fenofibrate, gemfibrozil, and bezafibrate), whereas agents such as thiazolidinediones, e.g., pioglitazone and others, are mainly PPARγ activators [27]. Some agents such as saroglitazar are dual activators of PPARα and γ [28] and elafibranor of both PPARα and β/δ [29,30]. Recently, very interesting natural compounds have been identified. Among these, arjunolic acid, a triterpenoid saponin, isolated earlier from *Terminalia arjuna* and later from *Combretum nelsonii*, *Leandra chaeton*, *Cochlospermum tinctorium*, and *Cornus capitata* is a powerful PPARα activator shown to regress cardiac fibrosis by inhibiting non-canonical TFG-1β signaling [31].

The activity of PPARα in controlling uptake and catabolism of fatty acids plays a critical role in generating fuel in the muscle and heart. It is mediated by the activation of mitochondrial carnitine palmitoyl transferase I (CPT1) [32], and responsible for the transfer of activated fatty acids across the mitochondrial outer membrane. In addition to fatty acid oxidation, the activity of PPARα includes a rise of apolipoprotein (apo)A-I, apoA-II, and ATP binding cassette (ABC)A1, and of HDL-C. There is an increase of very-low density lipoprotein (VLDL) clearance and activity of lipoprotein lipase (LPL) [33], with a concomitant reduction of apoC-III, VLDL production, and an increase of LDL particle size. The reduction of TGs and the increase of HDL after fibrates is fundamental in the management of the CV risk in central obesity associated with insulin resistance, which leads to a potential improvement of PPARα-related metabolic response [34].

Lastly, the development of the selective PPARα agonist, i.e., pemafibrate, offers a new dawn in the approach to address the treatment gap related to atherogenic dyslipidemia. Pemafibrate (formerly known as K-877) exhibits greatly enhanced PPARα potency and selectivity in cell-based transactivation assays, being > 2500-fold more potent than fenofibric acid, the active metabolite of fenofibrate, and > 5000-fold more specific for human PPARα than either PPARγ or δ [35].

## 4. Single Nucleotides Polymorphisms (SNPs)

SNPs, i.e., substitutions of a single nucleotide occurring at a specific position in the genome, are not rare with *PPARα* and each variant can be present to an appreciable degree within a population (i.e., >1%) [36]. A number of major PPARα SNPs have been detected [37].

Among the most relevant SNPs, one codes for an amino acid change. The rs1800206 C > G (L162V) is related to the involvement of PPARα in lipids and CV disease, being linked to the modulation of *PPARα* gene expression. The frequent SNP rs4253778 G > C (intron 7 G/C) is, instead, linked to the regulation of inflammation and oxidative stress [38].

The L162V polymorphism (rs1800206) is the only PPARα functional polymorphism found in the Caucasian population. It is consequent to a C-to-G transversion, which determines a leucine-to-valine substitution in the DNA binding domain of the human PPARα [39]. The transversion occurs at position 484 of exon 5 of the human *PPARα* gene, which encodes the second zinc finger of the DNA binding domain, adjacent to a cysteine essential to the coordination for the Zn^2+^ atom of the second zinc finger and immediately upstream of a region that determines the specificity and polarity of PPARα binding to DNA [40]. The valine allele generally encodes for a more active PPARα, particularly in conditions of high concentrations of a synthetic ligand [41].

This polymorphism has important effects in dyslipidemia. The V162 minor allele is associated with raised levels of total cholesterol, LDL-C, and triglycerides, thus leading to hyperlipidemia and to an increased CV risk in whites. This is more likely in non-diabetics than in diabetics [42,43], with the case being particularly strong in apoE4 carriers [44]. A gender difference was also noted in a Lithuanian population in which the impact of the CG genotype on TG rise [Odds Ratio (OR) = 2.67, 95% CI 1.15–6.16] was found only in men [45]. In the Framingham Offspring Study, the V162 in males was associated with significantly raised total and LDL-cholesterol and apo B levels. In females, a similar trend was observed but only the apoB rise was statistically significant [46]. Similar findings, in particular hyperapobetalipoproteinemia, in both genders, were reported in a Canadian study [43].

The 162Leu/Val genotype is more frequent in the German, Danish, Czech, and Brazilian population, whereas the 162Val/Val is more frequent only in the Tunisian population [47]. It is possible to demonstrate a much stronger association between this polymorphism (V162 allele), than the intron 7 G/C polymorphisms with stage C heart failure. The minor allele induces a reduced myocardial expression of long-chain 3-hydroxyacyl-CoA dehydrogenase and medium-chain acyl-CoA dehydrogenase [48] (Table 1).

The concentration of PPARα ligands influences the L162V polymorphism: V162 activity is almost half the L162 activity when PPARα ligands are either absent or at low concentrations, but it rises much more than the L162 if there are high concentrations of PPARα agonists [41]. This case could be of interest in order to use polyunsaturated fatty acids or fibrates to treat left ventricular hypertrophy or heart failure in V162 patients [49].

An association between the V162 allele and inflammation was reported by Caron-Duval in normal Canadians [50]. Carriers of the minor allele exhibited increased C-reactive protein (CRP) values after n-3 PUFA supplementation, vs a reduction in the L162 allele carriers, with no difference in the hypotriglyceridemic response. High-sensitivity CRP (hs-CRP) represents a major biomarker of inflammation and associated risk in CVD. This elevation can be corrected by few lipid lowering drugs [51,52]. This finding points out to a potentially elevated CV risk of the V162 carriers. Supporting this tentative conclusion is the reported association between the V162 allele and triglyceridemia in a large population sample in the United States [53]. V162 carriers has 78% higher TG levels with a 30% lower HDL-C, with this single polymorphism accounting for 3.8% of the variation in triglyceridemia. The additional effect of this polymorphism on adiposity points out to a potential impact on CV risk (Table 1).

A further support to the axioma linking the L162V polymorphism to the CV inflammatory burden is the detection, in a Chinese population, of an association between the minor V162 allele and lipoprotein (a) (Lp(a)) levels [54]. Compared with LL carriers, significantly higher Lp(a) concentrations were found in LV + VV individuals (mean difference: 49.07 mg/l, 95% CI 23.32–74.82 mg/l, *p* = 0.0002). People with the V allele had overall significantly higher Lp(a) levels than those with the L allele. Lp(a) likely contributes to CVD risk being more atherogenic than LDL since it contains both the proatherogenic components of LDL and the oxidized phospholipids [55]. However, these findings should be rated in the context of ethnic influences on the effects of the (L162V) variant. Shin et al. reported an association between the variant and an intronic SNP in the *PPARα* with plasma levels of apoC-III and TG in African-Americans versus no associations between these two SNPs and plasma levels of apoC-III and TG in Caucasians [56]. ApoC-III has been proposed as a prominent negative regulator of TG catabolism [57].

The effect of *PPARα* polymorphisms on diabetes is controversial. A *post-hoc* analysis of the STOP-NIDDM Trial in which patients with impaired glucose tolerance were randomized to either acarbose or placebo [58], reported that, in the placebo group, the G (V162) allele increased the risk of diabetes by 93% (95% CI 1.05–3.58), which is a feature associated with elevated levels of plasma glucose and insulin. Among patients allocated to the acarbose group, a higher diabetes risk was found in those carrying the minor G allele of rs4253776 (OR: 1.73, 95% CI 1.04–2.88) and the CC genotype of rs4253778 (OR: 2.78, 95% CI 1.14–6.79) [59]. Flavell et al., after evaluating three gene polymorphisms (an A > C variant in intron 1, the L162V variant, and the intron 7 G > C variant) and age at diagnosis in 912 Caucasian type 2 diabetic subjects, reported that the combination of the rare alleles of both the intron 1 A > C and intron 7 G > C variants synergistically lowered the age at diagnosis [60]. These findings were not supported by Silbernagel et al., who reported instead no association between the L162V SNP, type 2 diabetes, (body mass index) BMI, or body fat composition or liver fat content, concluding for a lack of impact of L162V on the pathogenesis of type 2 diabetes or obesity [61] (Table 1). Similarly, Sparsø et al. who genotyped the L162V polymorphism in the *PPARα* gene in 5,799 middle-aged white people, did not detect any association of the minor allele with obesity or type 2 diabetes [62].

The rs4253778 polymorphism is characterized by a G-to-C transversion at nucleotide 2528 of intron 7 in the human *PPAR*α gene, in the noncoding region. Even if nonfunctional, it affects PPARα transcriptional activity particularly in exercising and hypertensive subjects [49]. This polymorphism is linked to CAD risk, which is in partial allelic association with the L162V variant, and it shows opposite effects for CV risk [63]. The CC homozygotes show significantly higher total and LDL-cholesterol levels, not accounting, however, for the raised CV risk [63]. Contrasting findings were reported in a Brazilian case series of elderly individuals (mean 80 yo) where C carriers showed significantly lower TG and VLDL levels and higher HDL-C [64]. The association of CAD risk and dyslipidemia with the presence of the C allele was confirmed in an Indian population: the incidence of this polymorphism in CAD patients was 17.3% against 6.7% in the control group [65]. This polymorphism has been examined in a study on the progression of atherosclerosis in participants of the angiographic LOCAT and Northwick Park Heart studies [66], which confirm the link between increased risk of ischemic heart disease, progression of atherosclerosis, and C allele carrier status. In both of these studies, the V162 was associated with a delayed progression. The C-allele carrier status also appears to be associated with the development of left ventricular hypertrophy [49], especially in response to exercise and hypertension. Among patients who survived myocardial infarction, the carriers of the CC genotype showed higher levels of fetuin-A [67], a hepatokine associated with an increased CV risk [68,69] (Table 1).

Another coding SNP (rs1800234) is located in the hinge region of the *PPARα* gene. It leads to the sequence polymorphism Val227Ala. It is relatively frequent in the Oriental populations [70,71], and essentially absent in the West. This variant attenuates the transcription of cytochrome P-450 4A6 (35–56%) and mitochondrial 3HMG-CoA synthase genes in the presence of fibrate ligands [72]. This minor polymorphism is associated with reduced cholesterolemia and triglyceridemia, particularly in women, and appears to be associated with alcohol drinking habits. In more than 5% of the Japanese carriers, it appears to modify a response to alcohol. The A227 allele is associated with increased γ-glutamyl transpeptidase activity in drinkers. In non-drinkers, the V227 polymorphism leads to higher cholesterolemia, whereas, in drinkers, no significant lipid differences are found, which suggests that the V227 variant influences PPARα activity specifically in non-drinkers [73].

The coding Val227Ala SNP may be implicated in the pathogenesis of NAFLD, the latter being considered the hepatic manifestation of the MetS [74]. In 79 Chinese NAFLD patients and 63 healthy controls, the minor A227 allele frequency was significantly different between NAFLD and control subjects. A227 carriers had lower fat-related indexes, such as weight, BMI, hip circumference, waist circumference, waist-to-hip ratio, and the percentage of body fat [75]. In 401 healthy Japanese subjects, total cholesterol was lower in A227 carriers than in noncarriers, and the lipid profiles of A227 carriers appeared favorable compared to non-carriers [70]. Since the Val227Ala variant is located in the region between the DBD and LBD, which are also believed to contain the dimerization domain of the protein, it has been hypothesized that the substitution of A for V at codon 227 may cause a functional change. Consequently, the A227 isoform has higher activity than the V227 isoform [70], which potentially explains the association with lower lipids and protection from steatosis development [76].

Apart from the case of the V227A polymorphism, the L162V SNP does not appear to be linked to the risk of NAFLD. The frequency of this SNP in NAFLD was evaluated in 202 Italian subjects compared to 346 healthy controls. No significantly different incidence was found between patients and controls, but the V162 minor allele was associated with higher insulin resistance (IR) without histological disease severity, which suggests that the risk related to increased IR may be balanced by the protective effect of decreased oxidative stress, known as the other key player in the progression of liver disease [77] (Table 1).

Other areas of human disease have been explored in order to assess a possible connection with *PPARα* polymorphisms. The non-coding SNP rs4253747 appears to be associated with high altitude appetite loss in Chinese young men, with the A allele specifically associated with an increase in appetite loss (A vs T, OR = 1.79 95% CI = 1.08–2.95, *p* = 0.022). High altitude appetite loss is a common symptom of acute mountain sickness [78], with a complex physiological and genetic regulation [79]. It is hypothesized that the effect of PPARα on appetite could be mediated by the generation of OEA by epithelial cells in the small intestine, after fatty acid ingestion [80].

## 5. Interaction between *PPARα* Polymorphisms and Diet on Plasma Lipoproteins

Environmental factors such as the diet may interact with the genetic background to modulate metabolic parameters. The interaction between the L162V polymorphism and consumption of saturated and polyunsaturated fatty acids (PUFA) appears to affect lipid responses. In 1,023 male and 1,103 female participants in the Framingham study, the V162 allele was associated with higher plasma TG and apoC-III levels in subjects on a low PUFA diet (<6% of energy). The opposite was found when the PUFA intake was higher, which indicates a significant dose-response relationship between PUFA intake and TG concentrations, depending on the genotype [81]. In a study in healthy white men from Quebec, the carriers of the V162 allele had lower total cholesterol and apoA-I concentrations after a high PUFA diet [82] (Table 2).

There are, however, significant ethnic differences in the response to genetic variations in PPARα, as demonstrated by the 3′ untranslated region (UTR) SNPs (rs6008259 and rs3892755). Among participants from the ARIC (The Atherosclerosis Risk in Communities) study, these SNPs modulate the association between lipids and dietary intake of n-6 fatty acid (in whites) and long-chain n-3 fatty acids (in African Americans). Among those with this last heritage, carriers of either the *PPAR*α 3′UTR CC or CT genotypes, a larger intake of long-chain n-3 fatty acids (eicosapentaenoic acid + docosahexaenoic acid) was associated with higher levels of total cholesterol and LDL-C. The same trend was found in whites carrying either the *PPARα* 3′UTR GG or AG genotypes who consumed large amounts of n-6 fatty acids (>7.99 g/d). The opposite was found when the TT and AA genotypes were considered in African-Americans and whites, respectively [83] (Table 2).

## 6. Impact of *PPAR*α Polymorphisms on Fenofibrate Response

Changes in TG following fibrate treatment appear to be regulated by PPARα polymorphisms, as shown in the Diabetes Atherosclerosis Intervention Study. Among type 2 diabetic patients given fenofibrate, intron 7 G/G genotype (rs4253778) was more frequent in better responders (85% *vs* 69%). The G/G homozygosity proved be a significant predictor of the TG response (OR: 3.10, 95% CI 1.28–7.52) and, in those with raised baseline TG levels (>267 mg/dL), this SNP led to a relative reduction of >30% after fenofibrate [84]. Effects of rare, potentially functional variants of PPARα were reported in the GOLDN study (Genetics of Lipid Lowering Drugs and Diet Network) [85]. Of the 73 described variants with an average of 4.8% minor allele frequency, 13 were found to be associated with a reduced fenofibrate response (Table 3). In the group of extreme responders, carriers of at least one rare variant showed a low TG response after three weeks of fenofibrate treatment, with no effects on HDL-C, LDL-C, or inflammatory biomarkers, i.e., interleukin-6, interleukin-2, CRP, monocyte chemotactic protein-1, or tumor necrosis factor-α [85]. Studies on the L162V polymorphism in patients with different types of lipid disorders have failed to disclose any difference in response to fenofibrate treatment [34].

## 7. PPARα in Environmental Reprogramming of Metabolism

PPARα activity is modulated by post-translational modifications including phosphorylation, SUMOylation, ubiquitination, acetylation, and O-GlcNAcylation, that can be found at numerous modification sites [86], and all have a direct impact on cellular metabolism and energy production. PPARα, particularly in the liver, can be regulated by epigenetic modifications that can also induce significant changes. The possible regulation of epigenetic inheritance systems by environmental conditions may be crucial in the transfer of information to offspring [87].

Maternal diet, e.g., with protein restrictions throughout pregnancy, may lead to lower PPARα gene methylation, which may result in a 10.5-fold higher expression in the liver of the offspring compared to dams fed a control diet [88]. Epigenetic modifications on the specific expression pattern of PPARα target genes may be detected when following the long term history of a patient, as well in the case of heart failure patients, where changes in the direct methylation of DNA in the promoter region may cause silencing, particularly in PPARα-regulated energy genes [89].

## 8. Conclusions

Studies on the role of PPARα in major human tissues and, particularly, in liver, have indicated that mRNA expression is quite similar in humans and mice and may be reduced in severe hepatic disorders, such as non-alcoholic steatohepatitis and hepatitis C. In human liver, PPARα plays a pivotal role in various metabolic processes and its activation does not promote tumor formation, as found in rodents. Therefore, PPARα activators hold full promise as therapy for patients with obesity and type 2 diabetes, but also for subjects with severe liver conditions, such as NAFLD.

The activity of PPARα appears to be linked to its genetic variants. SNPs, in most cases, are related to non-coding variants with the exception of the L162V missense mutation in the Western regions and hinge region mutation V227A mainly in the East. While the L162V appears to be best linked to dyslipidemia, the hinge region mutation V227A appears to be more clearly linked to life habits (alcohol) and to sex-linked lipid abnormalities.

The predictive potential of these polymorphisms is certainly of value in improving our understanding of human disease, and also explaining variability in the clinical response to specific drug treatments or dietary approaches.

## Figures and Tables

**Table 1:** Single nucleotide polymorphisms of *PPARα*, location, and their potential interaction with biochemical findings.

SNP	Biochemical and Pathological Changes
rs1800206 C > G (Leu162Val) chr22:46218377 (GRCh38.p12)	Minor allele: (1) Increased cardiovascular disease risk *Rise in*: Total cholesterol, LDL-cholesterol, Triglycerides, C-reactive protein, Lipoprotein (a) and Apolipoprotein C-III *Decrement in*: HDL-cholesterol (2) Uncertain impact on diabetes development (3) No association with NAFLD
rs4253776 A > G (intron variant) chr22:46233582 (GRCh38.p12)	(1) Increased risk of diabetes
rs4253778 G > C (intron 7, G2528C) chr22:46234737 (GRCh38.p12)	Minor allele: (1) Increased cardiovascular risk factors (2) Increased risk of ischemic heart disease (3) Associated with the development of left ventricular hypertrophy in response to exercise and hypertension (4) Increased risk of diabetes (5) Increased levels of Fetuin-A
rs1800234 T > C (Val227Ala) chr22:46219983 (GRCh38.p12)	Minor allele: (1) Reduced cholesterolemia and triglyceridemia, particularly in women (2) Influence on alcohol drinking habits (3) Lower fat-related indexes
rs4253747 A > T (intron) chr22:46217340 (GRCh38.p12)	(1) High altitude appetite loss, common symptom of acute mountain sickness

**Table 2:** Interaction between *PPARα* polymorphisms and diet on plasma lipoproteins.

SNP	Lipid Changes
rs1800206 C > G (Leu162Val) chr22:46218377 (GRCh38.p12)	(1) In 162V allele carriers, low PUFA diet was associated with higher plasma TG and apoC-III levels. PUFA intake >8% corresponded to a 4% lower plasma TG (2) Lower total cholesterol and apoA-I upon a high PUFA diet
rs6008259 3′UTR G > A	(1) According to n-6 fatty acid intake (low vs. high consumers), in carriers of GG or AG genotypes, high daily intake (>7.99 g/d) of linoleic acid led to higher levels of total- and LDL-C
rs3892755 3′UTR C > T	(1) According to n-3 fatty acid intake (low vs. high consumers), in carriers of CC or CT genotypes, high daily intake (>0.32 g/d) of eicosapentaenoic acid + docosahexaenoic acid led to higher levels of total- and LDL-C

**Table 3:** *PPAR*α polymorphisms and difference in fenofibrate response.

SNP	Lipid Changes
rs4253778 G > C (intron 7; G2528C) chr22:46234737 (GRCh38.p12)	(1) In patients with type 2 diabetes, the intron 7 G/G genotype was a significant predictor of TG response (OR: 3.10, 95% CI 1.28–7.52)
13 rare variants (minor allele frequency < 1%): - intronic variant 1 (35 base pairs upstream of exon 2) - rs4253793 (5′–UTR, in the first half of exon 3) - synonymous mutation (base pair location 44972983) - rs1042311 (missense mutation (alanine to valine) located in exon 7) - The remaining nine of the 13 rare variants were located in the 3′ UTR of PPAR. This region often contains sequences targeted by microRNAs	(1) Decreased TG response after three weeks of fenofibrate
